# Practice of key essential nutrition action messages and associated factors among mothers of children aged six months to two years old in Karat town, Konso zone, South Ethiopia, 2024: a community-based cross-sectional study

**DOI:** 10.3389/fpubh.2024.1422203

**Published:** 2024-07-18

**Authors:** Tsehaynew Kasse, Zeleke Aschalew, Nathan Desalegn, Zenebe Jebero, Fikre Moga, Addisalem Haile

**Affiliations:** College of Medicine and Health Sciences, Arba Minch University, Arba Minch, Ethiopia

**Keywords:** essential nutrition action, practice, mothers, children, Konso zone, Ethiopia

## Abstract

**Background:**

Key essential nutrition actions (ENA) messages are a comprehensive and evidence-based nutritional package designed to improve the nutritional status during the critical first 1,000 days of life. The poor practice of ENA contributes significantly to mortality and morbidity related to malnutrition in young children. However, there is a dearth of studies focusing on the practice of key ENA messages among mothers and the factors associated with their practice. Therefore, this study aimed to assess the practice of key ENA messages among mothers of children aged 6 months to 2 years in Karat town, Konso zone, South Ethiopia in 2024.

**Methods:**

A community-based cross-sectional study involving 421 mothers of children aged 6 months to 2 years was conducted in Karat town, Konso zone, South Ethiopia from January 15 to February 29, 2024. Respondents were chosen using computer-generated random numbers. A structured, pretested, and interviewer-administered questionnaire was used to collect data. Following coding and entry into EpiData 3.1, the data were exported to SPSS version 25 for analysis. Logistic regression (bivariate and multivariable) was employed to identify factors influencing mothers’ practice of key ENA messages, and statistical significance was set at *p* < 0.05 with a 95% confidence interval.

**Results:**

The study found that 47.6% (95% CI: 42.8, 52.42%) of mothers demonstrated good practices. Having secondary education or higher, institutional delivery, receiving nutritional counseling during antenatal care (ANC), receipt of postnatal care (PNC) services, having good knowledge, and having a good attitude towards ENA all increase the likelihood of good practice.

**Conclusion:**

This study emphasizes the need for multifaceted interventions to improve ENA practice among mothers residing in Karat town. To effectively address this issue, it is crucial to implement targeted education programs, strengthen postnatal care services, and nutritional counseling into routine antenatal care, promote institutional deliveries, and enhance awareness.

## Introduction

The first 1,000 days of a child’s life, spanning from conception through the initial 2 years, are a crucial period for growth and development, with profound implications for long-term health outcomes (1, 2). Studies indicate that the failure to provide essential nutrients during this period leads to developmental delays, stunted growth, cognitive impairments, and heightened risks of chronic diseases in adulthood (3, 4).

The essential nutrition actions (ENA) framework provides a comprehensive approach to addressing critical nutritional needs during this period. This framework emphasizes seven key areas: exclusive breastfeeding, complementary feeding, and nutritional care for sick children, nutrition during pregnancy and lactation, and prevention of vitamin A, iron, and iodine deficiencies for women and children (1, 2).

The significance of ENA extends beyond individual health as it directly contributes to universal health coverage and helps alleviate the substantial economic burden associated with addressing malnutrition, which is estimated to be around 3.5 trillion US dollars annually (5, 6). By focusing on ENA, we can also make significant progress towards achieving the United Nations’ sustainable development goals (SDGs 2 and 3) targets (7) and World Health Organization (WHO’s) 2025 global nutrition targets (8).

Notably, ENA has the potential to prevent over 2 million maternal and child deaths each year. Despite its undeniable significance, over half of the children worldwide still lack access to these essential life-saving interventions (9).

Globally, in 2021, nearly 5 million children under the age of five lost their lives (10). Among these tragic fatalities, Sub-Saharan Africa accounted for 55%, with nearly 2.4 million deaths occurring before the children reached their second birthday, and 1 million of these occurred in Sub-Saharan Africa (10, 11). Malnutrition-related issues contribute to nearly 45% of all under-five fatalities (12), and 25% of nutrition-related morbidity and mortality were a result of poor practice of ENA (8).

Inadequate young child feeding practices (IYCF) during the first 2 years of life alone contribute to 40% of child fatalities, which rises to 25–50% in middle-income and low-income countries (13–15). Ethiopia, in particular, faces a significant challenge with malnutrition. The 2019 Ethiopian Demographic and Health Survey (EDHS) revealed that 37% of children under the age of five suffer from stunting, with 12% classified as severely stunted. Despite recommendations from WHO, optimal feeding practices in Ethiopia remain below average, with only 59% of infants under 6 months being exclusively breastfed and a mere 14% meeting the minimum dietary diversity (16).

Maternal nutrition during pregnancy plays a crucial role in reducing the incidence of small-for-gestational-age babies by 21% and increasing the average birth weight by 41 g. However, nearly 27% of pregnant mothers in Ethiopia experience malnutrition (17). Micronutrient deficiencies, particularly iron, vitamin A, and iodine further exacerbate the situation. Iron deficiency anemia affects around 20% of preschoolers globally and 40% of women of reproductive age in Africa, with Ethiopia experiencing significant prevalence rates of 57% of children under the age of five and 40% of women of reproductive age groups (18–21).

Vitamin A deficiency has also become prevalent in Ethiopia, with 33.9% of children under the age of two and 29% of pregnant women being affected. This deficiency contributes to more than 1 million childhood deaths and over 1.5 million cases of childhood blindness worldwide (22, 23).

Additionally, iodine deficiency affects 75% of pregnant women and 30% of preschool children globally, with the highest prevalence in Africa (42%). In Ethiopia, over 39.9% of children are iodine deficient, and only 37% of households use adequately iodized salt (24, 25).

In South Ethiopia, as reported in the most recent EDHS, the prevalence of wasting and stunting among children under five is 6.3 and 36.4%, respectively. Alarmingly, only 37% of children aged 6–35 months receive vitamin A supplementation. The report also revealed that the median duration of exclusive breastfeeding is only 4 months, and a mere 9.3 and 43.9% of children meet the minimum dietary diversity and meal frequency, respectively (16).

Previous studies in Ethiopia highlighted poor ENA practices, with factors such as educational status, monthly income, parity, place of birth, utilization of postnatal care services, level of knowledge, and attitude significantly influencing maternal practices (26, 27).

However, these studies predominantly emphasized rural settings. Therefore, this study aimed to address this gap by assessing maternal practices in an urban area, Karat town, and determining the potential role of nutritional counseling during antenatal care (ANC) on mothers’ practices, which has not been the focus of previous studies.

## Methods and materials

### Study area and period

**Figure 1:**
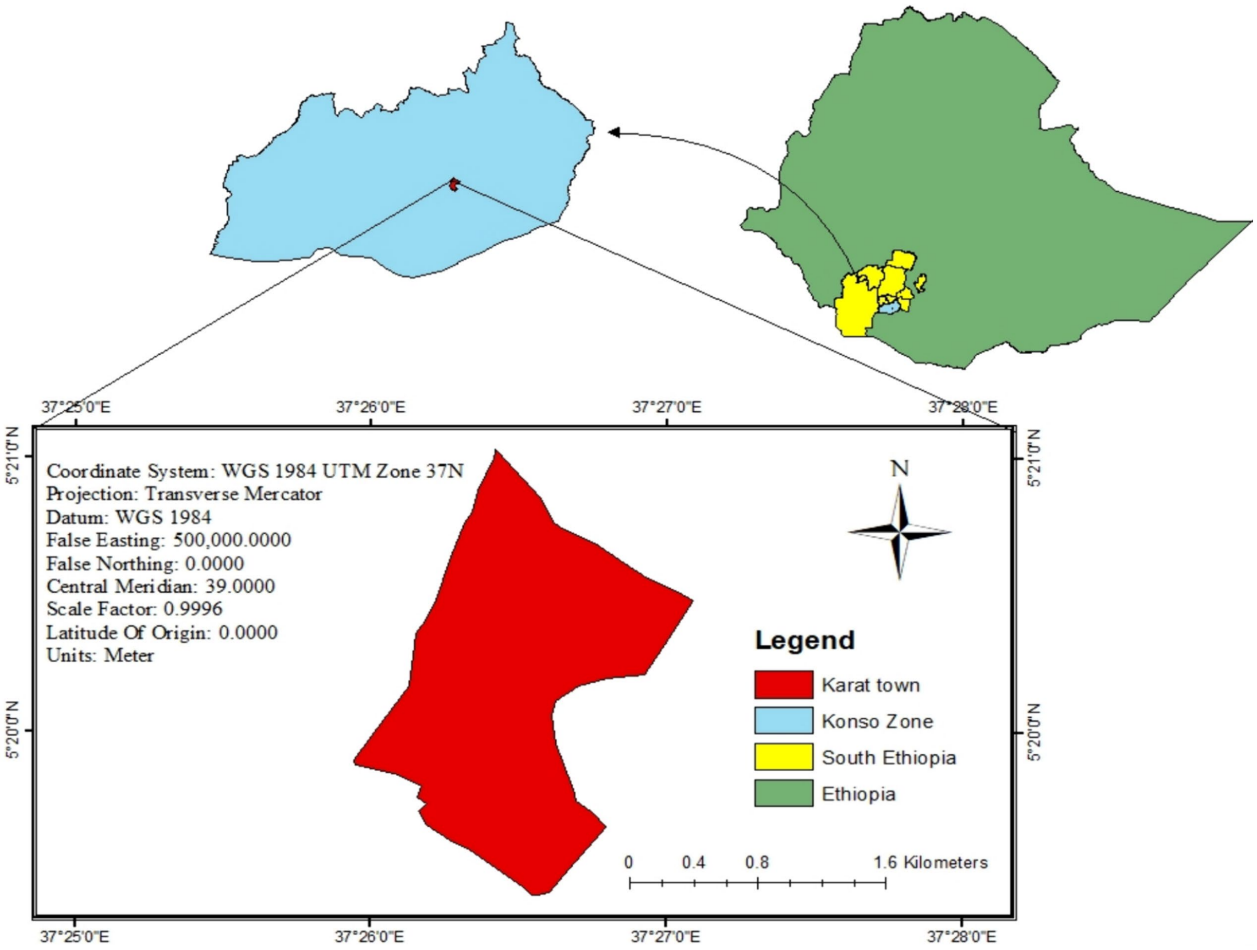
Geographic information system (GIS) representation of Karat town, Konso zone, South Ethiopia, 2024.

The study was conducted in Karat town, Konso Zone, South, Ethiopia. Karat is the capital town of Konso Zone, situated 607.2 km away from Addis Ababa. According to the information obtained from the town’s statistics office report (28), the total population of the town was 42,546, of whom 21,613 were female, 20,933 were male, 1,494 mothers had children from the age of 6 months to 2 years, and 8,676 households. There was 1 primary hospital and 1 health center with 7 health posts. Based on the health post they used, the town was divided into 7 administrative units (kebele) called Garisale, Dokatu, Dara paleta, Karate, Nalaya Segen, Etigele, and Gamole (Figure 1). The study was conducted from January 15 to February 29, 2024.

### Study design

A community-based cross-sectional study was conducted.

### Source population

All mothers of children aged from 6 months to 2 years old in Karat town.

### Study population

All mothers of children aged from 6 months to 2 years old in Karat town who fulfilled the inclusion criteria.

### Inclusion criteria and eligibility criteria

#### Inclusion criteria

All mothers who have children aged 6 months to 2 years, have lived in Karat town for more than 6 months, and have signed consent forms were included in the study.

#### Exclusion criteria

Mothers who had proven mental illnesses or were critically ill were excluded from the study.

### Sample size determination

The sample size of the study was determined using the single population proportion formula. Taking into account a 95% confidence interval (CI) and a 5% margin of error (d), along with a prevalence rate of 47.4% for the practice of key ENA messages among mothers from a study in Lemo District, Southern Ethiopia (27), and accounting in a 10% non-response rate, the final sample size was 421.

### Sampling technique and procedure

**Figure 2:**
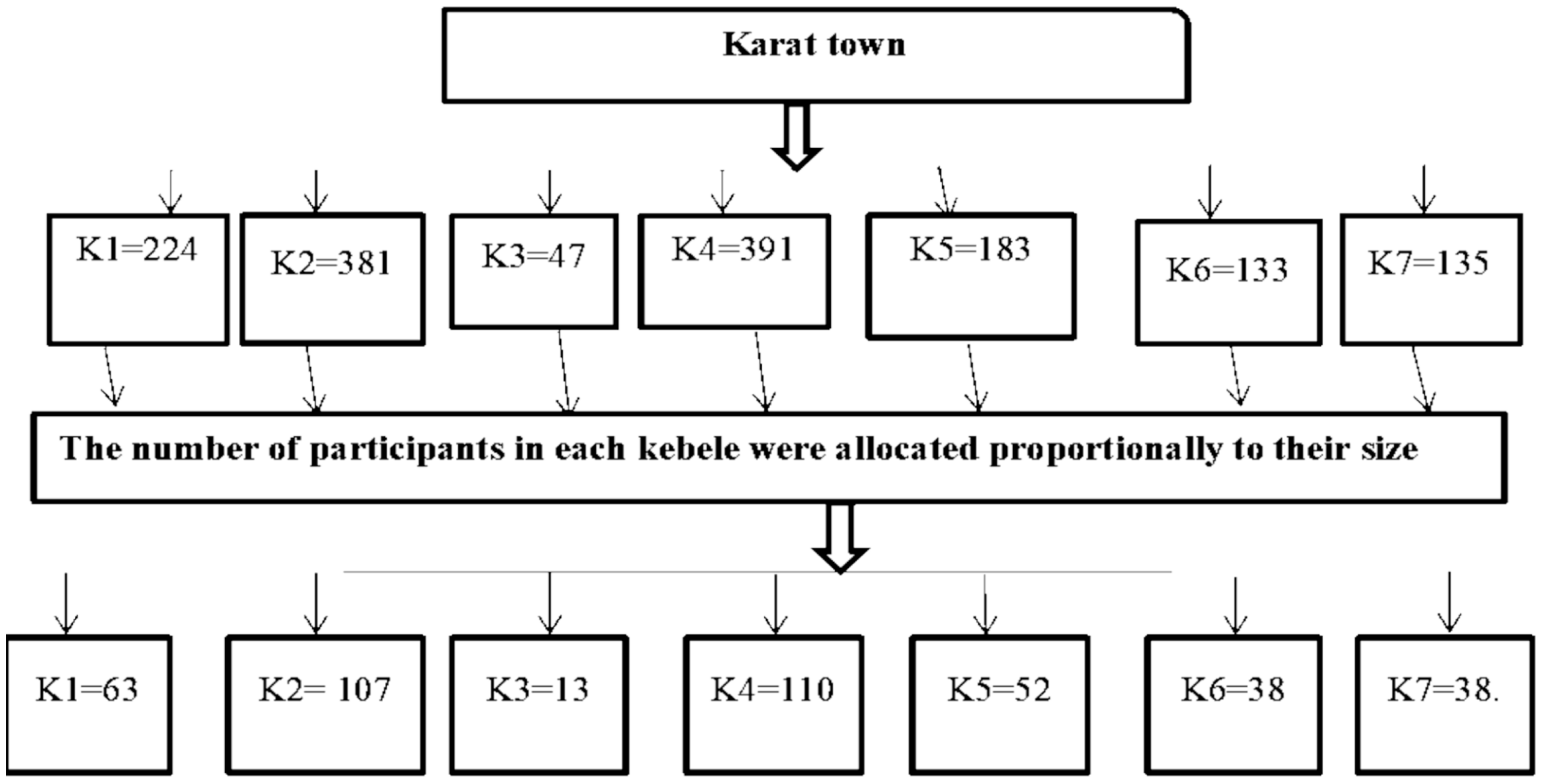
Schematic presentation of the sampling procedure used to assess the practice of key essential nutrition action messages and associated factors among mothers of children aged 6 months to 2 years in Karat town, Konso zone, South Ethiopia, 2024. The sampling included seven kebeles: K1 = Garisale Kebele, K2 = Dokatu Kebele, K3 = Dara paleta Kebele, K4 = Karate Kebele, K5 = Nalaya Segen Kebele, K6 = Etigele kebele, and K7 = Gamole Kebele.

A stratified random sampling technique was employed to select mothers with children aged 6 months to 2 years. The sample size was proportionally allocated to each kebele based on the total number of mothers with children aged 6 months to 2 years. The total number of mothers with children in the target age range (N) was determined (1,494). A proportional allocation factor (Nh) was calculated (n/N). This factor (0.282) was multiplied by the number of mothers in each kebele (ni) to determine the sample size allocated to each kebele (nh). To select the required sample, in each kebele, a computer-generated random number was used from the family folders registry, which was obtained from the health extension workers (Figure 2).

### Data collection instruments and procedures

Data collected for this study were obtained through a paper-based approach. A structured, pretested interviewer-administered questionnaire was used, which was adapted from previous studies (26, 27). Additionally, participants’ wealth status was evaluated using a tool consisting of 37 items adapted from EDHS (29). The questionnaire was divided into different sections, covering socio-demographic and economic factors which included eleven items (Section 1), maternal health service utilization which included three items (Section 2), mothers’ knowledge of ENA which included twenty-eight items (Section 3), mothers’ attitudes towards ENA messages included eighteen items (Section 4), and mothers’ practices related to key ENA which included twenty-seven items (Section 5).

The data collection team consisted of four diploma nurses who had previous experience in data collection. Additionally, two supervisors with Bachelor of Science degrees in nursing were recruited to oversee the study. To ensure the adequacy of the data collection tool, a pre-test was conducted on 21 mothers of children aged 6 months to 2 years in Karat zuria Woreda Fasha kebele 1 week before the actual data collection period. Based on the pretest experience, any ambiguities, confusion, or difficult words in the tool were revised. Furthermore, the internal consistency of the items in the knowledge, attitude, and practice sections was assessed using Cronbach’s alpha coefficients, resulting in values of 0.8, 0.79, and 0.88, respectively.

### Data quality control

To ensure quality, the questionnaire was initially developed in English and then translated into Amharic and Konsogna (local language), and then back to the English version by two independent language experts to assure consistency.

Appropriately designed and validated data collection tools were used, and data collectors and supervisors got 1 day of intensive training on the interview protocol, practice interviews, and discussions to address any questions or concerns. Supervisors and investigators maintained close oversight of the data collection processes daily. Investigators were checked for inconsistencies, and possible corrections were made during the data collection period. Study participants were interviewed in separate rooms in the house to reduce social desirability bias. To minimize the potential for recall bias, data collectors employed vitamin A, iron, and folic acid capsule samples during the data collection process. Additionally, respondents were provided with ample time to recall information accurately. Furthermore, study participants who were initially unavailable were revisited three times to ensure their inclusion in the study.

### Operational definition

Key ENA messages- A total of 27 items were used to assess the practice of key ENA messages and response categories for each practice assessment question were formed as “1 = correct response” and “0 = incorrect response”. A composite index of ENA practice was calculated, with zero indicating that women did not practice any ENAs and 27 indicating that those women practiced all ENA (26, 27).

#### Good practice

Those respondents who score mean and above the mean score of practice questions (26, 27).

#### Poor practice

Those respondents who scored below the mean score of practice questions (26, 27).

#### Knowledge of key ENA messages

A total of 28 items were used to assess the knowledge regarding ENAs and assessment questions were formed as “1 = correct response” and “0 = incorrect response”, and women who attained at least the mean score for the ENA knowledge assessment questions was labeled as having good knowledge, while those who did not will be labeled as poor knowledge (26, 27).

#### Attitudes of key ENA messages

A set of 18 items was used to evaluate attitudes using the Likert scale. Each item is assigned a score ranging from 1 to 3, where 1 indicates “not good,” 2 represents uncertainty or “not sure,” and 3 signifies “good.” Mothers who achieved a score equal to or higher than the mean score for the ENA attitude assessment questions were categorized as having a good attitude, while those who fell below the mean score were classified as having a poor attitude (26).

**Sick child-feeding practices** involved asking mothers about the frequency of feeding their children during illness. For children aged 6–8 months, the correct response was feeding more than 2–3 meals per day, while for those aged 9–23 months, it was more than 3–4 meals per day. On the other hand, mothers who provided the usual amount of liquids or less than usual, or withheld feeding, were classified as having poor sick baby-feeding practices (30).

#### Appropriate feeding

This means beginning breastfeeding within the first hour of birth, continuing to EBF for 6 months, and introducing semisolids, solids, and soft foods that are culturally appropriate from 6 months while breastfeeding is continued until 2 years and beyond, and the response was categorized as “yes” or “no” to each component (31).

#### Minimum meal frequency

Feeding 2–3 times per day for a child aged 6–8 months and 3–4 times per day for a 9–23 months aged child among breastfeeding mothers and at least 4 times per day among non-breastfeeding mothers, assessed by asking mothers the number of meals or feeds a child receives in the last 24 h before actual data collection (32, 33).

**Minimum dietary diversity** is the consumption of four or more food groups for higher quality, to meet the daily energy and nutrient requirements of the seven recommended food groups namely grains, roots, & tubers, legumes, and nuts, dairy products, flesh foods, fruits, and vegetables in the last 24 h prior actual data collection, responses were categorized as “yes” or “no” for each food group, and children who score at least four were considered to have met the requirement (34).

#### Wealth index

It is a composite measure of a household’s cumulative living standard. Based on the net score, the wealth status of respondents is classified into three. For a total of 37 items, including domestic animals, durable assets, productive assets, and dwelling characteristics, the response was categorized as “1 = yes” and “0 = no”, and any variable or assets owned by more than 90% or less than 5% were excluded. The Keiser-Mayer Olkin measure of sample adequacy (≥0.6) is used to check the principal component analysis (PCA) assumption, anti-image correlations (> 0.4), and Bartlett Sphericity Test (*p*-value 0.05) (27).

### Proper utilization of iodized salt

Adding salt to cooking at the end or right after cooking in the last 24 h (35).

#### Having postnatal care service

Mothers who attend at least one PNC service from health institutions by health professionals within 42 days of delivery (36).

### Data processing and analysis

After the data were collected, it was checked for completeness and coded before being entered. Then, the data were entered into epi data version 3.1 statistical software and then exported to Statistical Package for the Social Sciences (SPSS) version 25 for analysis. Descriptive statistics such as percentages, frequency, mean, and standard deviation was used to summarize the characteristics of the study participants and the findings were presented by using tables and graphs. The binary logistic regression model was fitted to identify factors associated with the practices of mothers towards key ENA. Initially, bivariable analysis was done to identify the candidate explanatory variables for the multivariable analysis. Thereafter, all explanatory variables having a *p*-value of less than 0.25 in the bivariable analysis were included in the multivariable logistic regression analysis to handle the effect of possible confounders and identify independent predictors of the practice of ENA messages in the final model. Model fitness for the final model was checked using Hosmer and Lemeshow goodness of fit (0.367). Multicollinearity was checked using the Variance Inflation Factor (VIF < 10). Adjusted odds ratio with 95% CI and a *p*-value of less than 0.05 were used to determine the level of significance.

## Result

### Socio-demographic characteristics of study participants

This study included a total of 418 mothers of children aged 6–24 months, resulting in a response rate of 99.3%. The mean age of the mothers was 25.66 years ±4.93.40.7% of them fell within the age range of 20–24 years, followed by those aged 25–29 (33%). The mean age of the children was 11.28 months ±3.9. Most of the respondents were married (89.7%), and 68.9% identified as followers of the Protestant Christian religion Additionally, 53.8% reported having a family size of four to five, while 42.3% had no formal education. A significant portion of the respondents were housewives (59.8%), around 34% had a medium household wealth index, and 37.1% reported that their husbands had no formal education (Table 1).

**Table 1:** Socio-demographic characteristics of mothers of children aged 6 months to 2 years old in Karat town, Konso zone, South Ethiopia, 2024.

Variable	Category	Frequency	Percentage
Age of the mother	<20	28	6.7
	20–24	170	40.7
	25–29	138	33
	30–34	57	13.6
	+35	25	6
Religion	Protestant	288	68.9
	Orthodox	76	18.2
	Muslim	32	7.7
	Catholic	22	5.3
Marital status	Married	375	89.7
	Single	16	3.8
	Windowed	14	3.3
	Divorced	13	3.1
Ethnicity	Konso	302	72.2
	Wolita	55	13.2
	Oromo	33	7.9
	Amhara	28	6.7
Educational status of a mother	No formal education	177	42.3
	Primary education	129	30.9
	Secondary	61	14.6
	Above secondary	51	12.2
Mothers occupation	Housewife	250	59.8
	Government employee	66	15.8
	Daily labor	16	3.8
	Merchant	22	5.3
	Private employee	64	15.3
Husband Education	No formal education	155	37.1
	Primary Education	123	29.4
	Secondary	81	19.4
	Above secondary	59	14.1
Family size	<4	114	27.3
	4–5	225	53.8
	6–8	63	15.1
	+9	16	3.8
Age of the child	6–8	98	23.4
	9–11	191	45.7
	12–24	129	30.9
Sex of the child	Male	239	57.2
	Female	179	42.8
Household wealth index	Poor	139	33.3
	Medium	140	33.5
	Rich	139	33.3

### Maternal and child health-related information of study participants

Among the study participants, more than half (63.2%) were multiparous, and the majority (82.5%) delivered their child to a healthcare facility. Furthermore, 65.3% of mothers received at least one postnatal service, and 59.1% of participants received nutritional counseling during antenatal care (ANC) (Table 2).

**Table 2:** Maternal and child health-related information of mothers of children aged 6 months to 2 years old in Karat town, Konso zone, South Ethiopia,2024.

Variable	Category	Frequency	Percent
Parity	Prim para	154	36.8
	Multi para	264	63.2
Place of delivery	Home	73	17.5
	Health institution	345	82.5
Having nutritional counseling during ANC	No	171	40.9
	Yes	247	59.1
Having PNC service	No	145	34.7
	Yes	273	65.3

Primi para = Having one viable pregnancy (≥ 28 weeks), Multi para = Having more than one viable pregnancy (≥ 28 weeks), ANC = Antenatal care, PNC = Postnatal care.

### Knowledge of respondents towards key ENA messages

**Figure 3:**
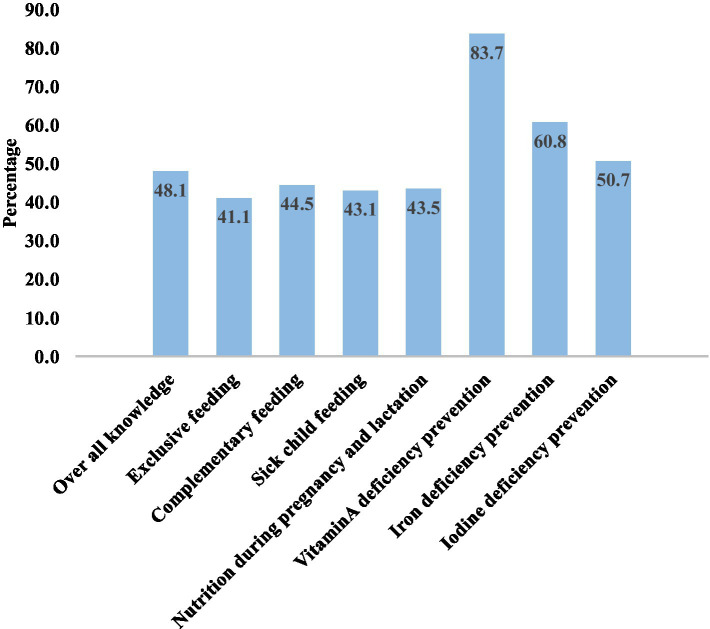
Good knowledge of respondents towards key ENA message in Karat town, Konso zone, South Ethiopia, 2024.

In Karat town, 48.1% (95% CI, 43.28 to 52.9%) of mothers of children aged 6 months to 2 years were knowledgeable about key essential nutrition action messages. Specifically, most of the (83.7%) mothers exhibited a strong understanding of preventing vitamin A deficiency, while 60.8% knew about preventing iron deficiency anemia. However, only 41.1% of mothers were knowledgeable about exclusive breastfeeding (Figure 3).

### Attitudes of the mother towards key ENA messages

In this study, 47.6% (95% CI, 42.8–52.42) of mothers had a good attitude regarding key essential nutrition action messages.

### The practice of respondents on key ENA messages

**Figure 4:**
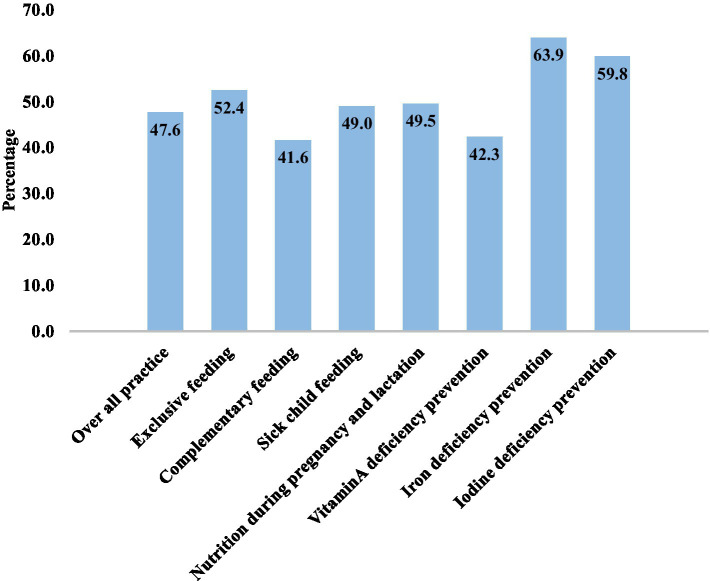
Good practice of key essential nutrition action messages among mothers of children aged 6 months to 2 years old in Karat town, Konso zone, South Ethiopia, 2024.

The overall practice of key ENA messages among mothers 47.6% (95% CI: 42.8, 52.42%) had good practice. Iron deficiency anemia prevention and iodine deficiency prevention were the most commonly practiced items by 63.9 and 59.8% of respondents, respectively. However, 41.6% of respondents had good practice in complementary feeding (Figure 4).

### Exclusive breastfeeding practice

In this study, 98.6% of mothers chose to breastfeed their children, with 59.1% initiating breastfeeding promptly. However, 24.2% of mothers practiced pre-lacteal feeding, 20.8% avoided feeding colostrum, and 32.5% resorted to bottle feeding (Table 3).

**Table 3:** Exclusive breastfeeding practice of mothers of children aged 6 months to 2 years old in Karat town, Konso zone, South Ethiopia, 2024.

Categories	Frequency	Percent
Have you ever breastfed	Yes = 412	98.6
	No = 6	1.4
Timely initiation of breastfeeding	Immediately = 247	59.1
	Hours = 171	40.9
Pre-lacteal feeding	Yes = 101	24.2
	No = 317	75.8
Colostrum avoidance	Yes = 87	20.8
	No =331	79.1
Bottle feeding	Yes = 136	32.5
	No = 282	67.5
Frequency of breastfeeding	Ten times &more =279	66.7
	Less than ten = 139	33.3

### Complementary feeding practice

The study revealed that a majority of participants (94.7%) introduced complementary feeding to their children. Of those 71.1% of them initiated at 6–8 months of age. Less than half of the mothers (44.7%) were able to meet the minimum meal frequency (MMF) requirements for their children, and only 25.8% managed to offer the minimum dietary diversity recommended for their children. Furthermore, 98.1% of mothers continued to give breastfeeding to their children.

### Sick child feeding practice

In this study, about half (50.5%) of study participants reported that their children had experienced illness in the 2 weeks preceding data collection. Among mothers of these children, 49% (95% CI: 44–53.8%) were found to exhibit good feeding practices with 73% providing more breast milk than usual, 19.9% offering the same amount as usual, and 7.1% giving lesser amount than usual to their sick children. Regarding fluid intake, 58.8% of mothers of sick children gave extra fluids, 27% gave the same amount as usual, and 14.2% gave less fluid than usual. Furthermore, concerning the quantity of food being provided, the majority (53.1%) of sick children were given more than the usual amount, followed by 36% who were provided with the same amount as usual, and 10.9% were given less than the usual amount.

Regarding the nutritional practice of respondents during their pregnancy and lactation period in the study, 56.9% of respondents reported having an additional meal. Notably, 84.2% of respondents included animal products in their meal variety.

### Practice of respondents on the prevention of vitamin a, iron deficiency anemia, and iodine deficiency

Less than half (45.7%) of mothers consumed Vitamin A supplements within 45 days of delivery, while 78% of their children received vitamin A supplementation at least once. In the 24 h dietary recall before the date of data collection, 59.8% of participants consumed green leafy vegetables, 39.2% consumed animal sources, and only 1% consumed ripe fruits like mango, from vitamin A-rich foodstuffs.

Regarding iron deficiency anemia prevention practice, three-fourths (74.4%) of participants reported taking iron/folic acid supplements. Furthermore, 81.1% of individuals included organ meats and other animal products in their diets. Additionally, a significant 81.1% of participants consumed green leafy vegetables.

Moreover, in terms of iodine deficiency prevention practice, only 31.1% of mothers used non-iodized salt for preparing meals. Of those who added salt to stew, 62.2% did so in the middle of the cooking process, while 37.8% added it at the end. Additionally, 62% of mothers stored salt in a closed container.

### The association of the gender of the child with maternal practice towards key essential nutrition action messages

The study found that there is no significant association between the gender of the child and maternal practice towards key essential nutrition action messages, with its Pearson chi-square test value (2.645) and its *p*-value of (0.104) (Table 4).

**Table 4:** The association of gender of the child with maternal practice towards key essential nutrition action in Karat town, Konso zone, South Ethiopia, 2024.

Gender of the child	Good practice (%)	Poor practice (%)	Chi-square value	df	*p*-value
Male	122 (61.3%)	117 (53.4%)	2.645	1	0.104
Female	77 (38.7%)	102 (46.6%)			

df, means degree of freedom.

### Factors associated with the practice of key ENA messages

In the bivariable analysis, factors such as the mother’s education level, the husband’s education level, the mother’s occupation, place of delivery, receiving nutritional counseling during ANC, receiving PNC services, knowledge, and attitudes towards key ENA messages were found to have statistical significance (*p* < 0.25). However, in the multivariable analysis, after considering all these factors together, the educational status of the mother, receiving nutritional counseling during ANC, receiving PNC services, place of delivery, knowledge, and attitudes towards key ENA messages were identified as statistically significant (*p* < 0.05).

The odds of practice approximately three times with Adjusted Odds Ratio (AOR: 2.82, 95% CI: 1.3–6.15) were higher among mothers with secondary education and about four times (AOR: 4.39, 95% CI: 1.8–10.69) higher among mothers with above secondary education than mothers with no formal education. In comparison to mothers who did not receive nutritional counseling during ANC, the odds of practice increased about three times (AOR: 2.51, 95% CI: 1.46–4.299) in those who received it. The odds of practice were 1.75 times more likely in mothers who receive postnatal care services when compared to their counterparts (AOR: 1.75, 95% CI:1.006–3.037), Mothers who deliver their child in a health institution were about two times more likely to practice than those who delivered at home (AOR: 2.31, 95% CI:1.17–4.56). Furthermore, mothers who had good knowledge and attitude towards key ENA were about three times (AOR: 3.47, 95% CI: 1.89–6.39) and two times (AOR: 2.06, 95% CI: 1.14–3.75) higher in practicing key ENA messages than those who had not, respectively (Table 5).

**Table 5:** Factors associated with the practice of key essential nutrition action messages among mothers of children aged 6 months to 2 years old in Karat town, Konso zone, South Ethiopia, 2024.

Variable	Categories	Mothers practice		COR(95% CI)	AOR = (95% CI)	*p*-value
		Good (%)	Poor (%)			
Educational status of a mother	No formal education	57 (28.6%)	120 (54.8%)	1	1	
	Primary	54 (27.1%)	75 (34.2%)	1.52 (0.95–2.45)	1.19 (0.67–2.12)	0.559
	Secondary	47 (23.6%)	14 (6.4%)	7.07 (3.6–13.9)	2.82 (1.3–6.15)*	0.009
	Above secondary	41 (20.6%)	10 (4.6%)	8.63 (4.04–18.5)	4.39 (1.8–10.69)*	0.001
Husbands educational status	No formal education	51 (25.6%)	104 (47.5%)	1	1	
	Primary	59 (29.6%)	64 (29.2%)	1.88 (1.16–3.06)	1.779 (0.92–3.44)	0.87
	Secondary	53 (26.6%)	28 (12.8%)	3.86 (2.19–6.81)	1.92 (0.92–3.995)	0.83
	Above secondary	36 (18.1)	23 (10.5%)	3.19 (1.72–5.94)	1.73 (0.77–3.87)	0.184
Mothers occupation	Housewife	115 (5.8%)	135 (61.6%)	1.095 (0.63–1.9)	1.06 (0.53–2.13)	0.868
	Government employee	34 (17.1%)	32 (14.6%)	1.37 (0.69–2.73)	1.24 (0.52–2.98)	0.626
	Daily labor	7 (3.5%)	9 (4.1%)	1.0 (0.33–3.02)	0.755 (0.21–2.78)	0.672
	Merchant	15 (7.5%)	7 (3.2%)	2.76 (0.99–7.67)	1.45 (0.41–5.15)	0.566
	Private employee	28 (14.1%)	36 (16.4%)	1	1	
Place of delivery	Home	19 (9.5%)	54 (24.7%)	1	1	
	Health institution	180 (90.5%)	165 (75.3%)	3.1 (1.76–5.45)	2.31 (1.17–4.56)*	0.016
Nutritional counseling during ANC	Yes	157 (78.9%)	90 (41.1%)	5.36 (3.47–8.27)	2.51 (1.46–4.299)**	<0.01
	No	42 (21.1%)	129 (58.9%)	1	1	
Having PNC service	Yes	162 (81.4%)	111 (50.7%)	4.26 (2.73–6.65)	1.75 (1.006–3.037)*	0.048
	No	37 (18.6%)	108 (49.3%)	1	1	
Mothers knowledge	Poor	50 (22.8%)	169 (77.2%)	1	1	
	Good	151 (75.9%)	48 (24.1%)	10.63 (6.76–16.7)	3.47 (1.89–6.39)**	<0.01
Mothers attitude	Poor	56 (25.6%)	163 (74.4%)	1	1	
	Good	143 (71.9%)	56 (28.1%)	7.43 (4.82–11.46)	2.06 (1.14–3.75)*	0.017

*p < 0.05, **p < 0.01; AOR, adjusted odds ratio; COR, crude odds ratio; CI, confidence interval.

## Discussion

This study aimed to assess the practice of key ENA messages and associated factors among mothers of children aged 6 months to 2 years in Karat town, Konso zone, South Ethiopia. According to the findings of this study, the overall magnitude of good ENA practice was 47.6% (95% CI: 42.8, 52.42%).

The result of this study is consistent with similar studies conducted in the Northeast (46.5%) (26) and Southern Ethiopia (47.4%) (27). However, it was lower compared to a study in Southwest Ethiopia (37). Possibly due to differences in the study population, the level of knowledge, and the attitude of respondents towards ENA.

The exclusive breastfeeding practice in this study was found to be 52.4%, which is higher than the rates reported in previous studies conducted in Addis Ababa (29.3%) (38) and Northern Ethiopia (30.7, 34.8%) (39, 40). However, it falls below rates reported in the Amhara (79%) (41) and Sidama regions of Ethiopia (60.9%) (42). These variations could be attributed to factors such as maternal employment status, and utilization of maternal and child health services.

Complementary feeding practices were found to be inadequate, with only 25.8% of children meeting the minimum dietary diversity requirement. Consistent with findings in a study in Eastern Ethiopia (24.4%) (43), and (27.3%) in Wolita (33), but higher than rates reported in rural settings studies in the Oromia region (16%) (44), and (12.6%) in Northwest Ethiopia (45). Conversely, higher rates were observed in urban areas such as Addis Ababa (59.9%) (34). This could be influenced by socio-economic factors and access to diverse food and nutrition awareness (46–48).

Regarding sick child feeding practices, 49% of mothers demonstrated good practices, which is consistent with studies conducted in Eastern Ethiopia (45%) (49), the Gamo zone in South Ethiopia (45%) (50), and Addis Ababa (54%) (30). However, it is lower than the study conducted in Mirab Abaya, South Ethiopia (70.7%) (51). The variations in results could be attributed to factors such as marital status, access to support for child feeding, information availability, and the educational background of the mothers.

In terms of nutritional practice during pregnancy and lactation, 56.6% of participants reported consuming an additional meal, a higher percentage compared to previous studies in Southern (36.9%) (52), and Northeast Ethiopia (43.9%) (53). However, it was lower than the study in the Oromia region (75.4%) (54). The observed variations in nutritional practices could be attributed to factors such as access and utilization of maternal and child health services, socio-economic status, and the educational background of the respondents.

In terms of the prevention of vitamin A deficiency practice, 45.7% of mothers reported taking vitamin A within 45 days of delivery. This is higher than 39.1% in a study conducted in Southern Ethiopia (27) The possible reason might be differences in the study areas, period, and socio-demographic characteristics. However, it is lower than 63.5% in the Amhara region (26). The disparities in uptake could be attributed to varying levels of knowledge and attitudes towards ENA among mothers. Additionally, 78% of children received vitamin A supplements at least once, which is notably higher compared to previous studies in the Sidama region (36.2%) (55), and (58%) in Southern Ethiopia (56). The rural settings and lower educational backgrounds of mothers in these studies may contribute to the differences.

Regarding the practice of mothers on iron deficiency anemia prevention, 74.4% of mothers took iron/folic acid during their recent pregnancy, which was found to be higher than rates reported in South and Eastern Ethiopia (50.06%) (57), Kenya (31.7%) (58) and Pakistan (38.3%) (59). The higher compliance could be linked to factors like urban residence, facility-based deliveries, and higher educational levels among participants.

Furthermore, regarding iodine deficiency prevention practice, 68.9% of participants reported utilizing iodized salt, which closely aligns with 72.2% in the Tigray region (60). However, it is higher compared to studies in Southeast Ethiopia (56.6%) (61), and the Somali region (26.6%) (62). The urban setting of this study and a higher proportion of mothers with primary education or above may contribute to better awareness and easier access to iodized salt.

Regarding factors associated with the practice of key ENA messages, mothers with secondary education or above are more likely to practice ENA, compared to mothers with no formal education. This finding is congruent with previous studies conducted in the Amhara region (26) and Southern Ethiopia (27). The increased awareness of health-related issues, including nutrition, among educated participants, positively influences their engagement in ENA practices. This, in turn, leads to better maternal and child nutrition outcomes (63, 64).

Another significant factor that influences ENA practices is the place of delivery. Delivery in health institutions increases twice the odds of practicing ENA, than those who gave birth at home. Which was found to be congruent with studies in Northern (26) and Southern Ethiopia (27). This could be attributed to the nutritional education and support provided by trained health professionals in health institutions, which leads to immediate benefits such as improved postnatal nutrition and reduced risk of malnutrition.

Mothers who received nutritional counseling during ANC were found to be three times more likely to engage in ENA practices compared to those who did not receive it. This finding is supported by a study conducted in Southwest Ethiopia (37), and Eastern Ethiopia (65). A possible explanation for this effect is that nutritional counseling provides valuable information about dietary requirements during pregnancy and lactation (66), increasing mothers’ knowledge about essential nutrition actions (67). Moreover, counseling facilitates behavioral changes related to healthier eating habits and lifestyle choices, as well as equipping mothers with information on where to access nutritious foods and supplements (68). The use of positive reinforcement techniques during counseling sessions further strengthens desired behaviors associated with ENA (69).

Moreover, receiving PNC services is also associated with higher odds of practicing ENA. This finding is consistent with studies in the Amhara region (26), and Southern Ethiopia (27). The reason behind this might be, that PNC sessions often include nutrition education, emphasizing exclusive and complementary feeding practices. This likely encourages mothers to adopt and adhere to ENA, which helps to address nutritional challenges for both mothers and children.

Additionally, attitude and knowledge emerged as other influencers, and the likelihood of practice increased twice among a mother who had a good attitude regarding ENA. This finding is concurrent with a previous study in the Amhara region (26). A possible explanation could be that a positive attitude towards a given behavior will lead to more practice of that behavior as evidenced by different research studies (70, 71), and by a well-known cognitive consistency theory (72).

Furthermore, mothers who had good knowledge were more likely to practice essential nutrition action. This is in agreement with a study in the Amhara region (26). The possible reason could be, that mothers with good knowledge are more likely to practice exclusive breastfeeding and more likely to consult healthcare professionals when they have concerns about their child’s nutrition, which can lead to better nutrition practices for their children (70, 73).

The study’s results have important clinical implications for enhancing dietary diversity, improving health education and awareness initiatives, empowering healthcare professionals to provide comprehensive postnatal care and nutritional counseling, advocating for institutional deliveries, and guiding the development of evidence-based policies and programs aimed at enhancing maternal and child nutrition in Ethiopia.

## Conclusion

This study revealed that the practice of key essential nutrition action messages among mothers was found to be poor. Factors such as having secondary education or higher, delivering in a health institution, receiving postnatal care services, and nutritional counseling during antenatal care, and possessing good knowledge and attitudes were significantly associated with good practice.

## Recommendation

The concerned body including governmental and non-governmental organizations should give due emphasis on maternal education for those with lower education levels, enhancing postnatal care services, strengthening nutritional counseling into routine ANC, and promoting institutional deliveries. Future research should focus on healthcare providers’ perspectives on delivering ENA messages and the role of social support networks in promoting optimal nutrition practices among mothers to uncover other influencing factors.

## Strength and limitation of the study

### Strengths

The study’s strengths lie in its focus on key ENA messages tailored specifically for mothers residing in urban areas, rather than solely focusing on rural settings. Additionally, the study’s ability to offer an accurate snapshot of maternal practices within this particular population without requiring long-term follow-up.

### Limitations

The cross-sectional nature of this study makes causal relationships between dependent and independent variables impossible. Since the study was based on self-reports, the respondents might be prone to social desirability bias. Finally, because women were asked about incidents that had already occurred before the study period, there may be a risk of recall bias.

## Data availability statement

The raw data supporting the conclusions of this article will be made available by the authors, without undue reservation.

## Ethics statement

Ethical clearance was obtained from the Institution’s Research Ethics Review Board of Arbaminch University (protocol No. IRB/1433/ 2023). The studies were conducted in accordance with the local legislation and institutional requirements. The participants provided their written informed consent to participate in this study.

## Author contributions

TK: Conceptualization, Data curation, Formal analysis, Funding acquisition, Investigation, Methodology, Project administration, Resources, Software, Supervision, Validation, Visualization, Writing – original draft, Writing – review & editing. ZA: Conceptualization, Data curation, Formal analysis, Funding acquisition, Investigation, Methodology, Project administration, Resources, Software, Supervision, Validation, Visualization, Writing – original draft, Writing – review & editing. ND: Conceptualization, Data curation, Formal analysis, Funding acquisition, Investigation, Methodology, Project administration, Resources, Software, Supervision, Validation, Visualization, Writing – original draft, Writing – review & editing. ZJ: Supervision, Validation, Visualization, Writing – original draft, Writing – review & editing, Conceptualization, Data curation, Formal analysis, Funding acquisition, Investigation, Methodology, Project administration, Resources, Software. FM: Conceptualization, Data curation, Formal analysis, Funding acquisition, Investigation, Methodology, Project administration, Resources, Software, Supervision, Validation, Visualization, Writing – original draft, Writing – review & editing. AH: Conceptualization, Data curation, Formal analysis, Funding acquisition, Investigation, Methodology, Project administration, Resources, Software, Supervision, Validation, Visualization, Writing – original draft, Writing – review & editing.
